# Regenerative Virtual Therapy: The Use of Multisensory Technologies and Mindful Attention for Updating the Altered Representations of the Bodily Self

**DOI:** 10.3389/fnsys.2021.749268

**Published:** 2021-11-03

**Authors:** Giuseppe Riva, Silvia Serino, Daniele Di Lernia, Francesco Pagnini

**Affiliations:** ^1^Applied Technology for Neuro-Psychology Laboratory, Istituto Auxologico Italiano, Milan, Italy; ^2^Humane Technology Laboratory, Università Cattolica del Sacro Cuore, Milan, Italy; ^3^Department of Psychology, Università Cattolica del Sacro Cuore, Milan, Italy; ^4^Department of Psychology, Harvard University, Cambridge, MA, United States

**Keywords:** embodiment (and its derivatives), multisensory integration, Bayesian surprise maximization, bodily full-body illusions, brain stimulation, interoceptive technology, virtual reality, mindfulness

## Abstract

The term “regenerative medicine” (RM) indicates an emerging trend in biomedical sciences that aims at replacing, engineering, or regenerating human cells, tissues, or organs to restore or establish normal function. So far, the focus of RM has been the physical body. Neuroscience, however, is now suggesting that mental disorders can be broadly characterized by a dysfunction in the way the brain computes and integrates the representations of the inner and outer body across time [bodily self-consciousness (BSC)]. In this perspective, we proposed a new kind of clinical intervention, i.e., “Regenerative Virtual Therapy” (RVT), which integrates knowledge from different disciplines, from neuroscience to computational psychiatry, to regenerate a distorted or faulty BSC. The main goal of RVT was to use technology-based somatic modification techniques to restructure the maladaptive bodily representations behind a pathological condition. Specifically, starting from a Bayesian model of our BSC (i.e., body matrix), we suggested the use of mindful attention, cognitive reappraisal, and brain stimulation techniques merged with high-rewarding and novel synthetic multisensory bodily experience (i.e., a virtual reality full-body illusion in sync with a low predictabIlity interoceptive modulation) to rewrite a faulty experience of the body and to regenerate the wellbeing of an individual. The use of RVT will also offer an unprecedented experimental overview of the dynamics of our bodily representations, allowing the reverse-engineering of their functioning for hacking them using advanced technologies.

## Introduction

Holmes et al. (2014) published, in Nature, the article “Psychological treatments: A call for mental-health science,” calling for an alliance between clinicians and neuroscientists to advance our understanding of psychological treatments. They underlined that “we do not yet fully understand how psychological therapies work—or when they don’t. Neuroscience is shedding light on how to modulate emotion and memory, habit, and fear learning. But psychological understanding and treatments have, as yet, profited much too little from such developments.” (p. 288) A key problem underlying most, if not all, psychopathologies is schema rigidity (Morris and Mansell, 2018): many individuals are unable to avoid and update automatic beliefs and behaviors that rely on preexisting or underlying assumptions and evaluations that might not apply to the current situation with significant negative effects.

However, recent key discoveries in neuroscience are outlining a new conceptual framework, merging the embodied cognition approach (Clark, 2016b; Newen, 2018) with the predictive brain hypothesis (Friston, 2010; Owens et al., 2018), on how self-schemas influence the psychological functioning that directly links them to the processing of multisensory bodily signals (Blanke et al., 2015; Riva, 2018). Paulus et al. (2019) recently explained that “these conceptual models suggest that mental disorders can be broadly characterized by a dysfunction in the way the brain computes and integrates representations of the inner and outer worlds of the body across time. According to this view, changes in mood and anxiety are a by-product of the brain’s biased translation of what it expects will happen versus what is actually happening in these worlds, producing a persistent discrepancy/error signal when outcomes are observed.” (p. 99).

Following this vision, in the last decade, several mental health conditions have been associated with damage and/or malfunctioning of the bodily self, i.e., eating and weight disorders (Riva and Gaudio, 2012; Keizer et al., 2013; Dakanalis et al., 2016; Scarpina et al., 2016; Riva and Dakanalis, 2018), depression (Barrett et al., 2016), schizophrenia (Postmes et al., 2014; Klaver and Dijkerman, 2016; Ferri et al., 2017; Möller et al., 2021), autism (Ropar et al., 2018; Riva et al., 2019a), and chronic pain (Tsay et al., 2015; Di Lernia et al., 2016).

Nevertheless, since the study by Holmes et al. (2014), things have not changed significantly: these basic research discoveries have not yet met a direct clinical application. While the change mechanisms of successful psychotherapeutic approaches, such as the current gold standard for many mental diseases, i.e., cognitive behavioral therapy (CBT), are often based on schema modifications, they do not target directly with their methods all the components of a faulty bodily experience.

In 2014, the first author of this perspective suggested in a letter to Nature (Gaggioli and Riva, 2014) that the use of technology, and in particular virtual reality (Riva et al., 2019b), could be a possible solution to this problem, offering a powerful tool for improving evidence-based psychological treatments. More recently, the two different studies by Browning et al. (2020) and Nair et al. (2020) suggested the use of computational characterizations/assays of behavior for patients undergoing psychological therapies using mathematical/Bayesian models of key cognitive processes.

In this perspective, we wanted to follow both suggestions by introducing a new therapeutical approach, i.e., Regenerative Virtual Therapy (RVT). Specifically, starting from a Bayesian model of our bodily self (i.e., body matrix), we suggested the use of mindful attention, cognitive reappraisal, and brain stimulation techniques merged with high-rewarding and novel synthetic multisensory bodily experience to rewrite a faulty bodily experience and to regenerate the wellbeing of an individual.

## From Regenerative Medicine to Regenerative Virtual Therapy

In medicine, a profound paradigm shift was introduced by regenerative medicine (RM), an emerging trend in biomedical sciences that aims at “replacing, engineering, or regenerating human cells, tissues, or organs to restore or establish normal function” (Mason and Dunnill, 2008). The fundamental value of RM is the possibility to regenerate the organism and to force the body to heal itself. RM allows not only to better cope with the symptoms but also to eradicate the cause of the symptoms by helping the body to restore the damaged cells to a healthy state (Mason and Dunnill, 2008; Mahla, 2016).

So far, the focus of RM has been the physical body: human stem cells and biomolecular therapies are used to restore the normal structure and function of a missing or damaged organ. However, the abovementioned evidence from recent neuroscientific discoveries suggests that by exploiting the mechanisms of the “predictive brain,” it is also possible to regenerate our bodily experience [i.e., bodily self-consciousness (BSC)].

The BSC represents a challenging research field because it requires an interdisciplinary framework to provide a link between all the afferent levels and brain circuits involved in a particular bodily experience (Lux et al., 2021). However, recent neuroscience research (Blanke, 2012; Blanke et al., 2015; Riva, 2018; Park and Blanke, 2019) is shedding new light on the processes involved in the BSC.

**FIGURE 1:**
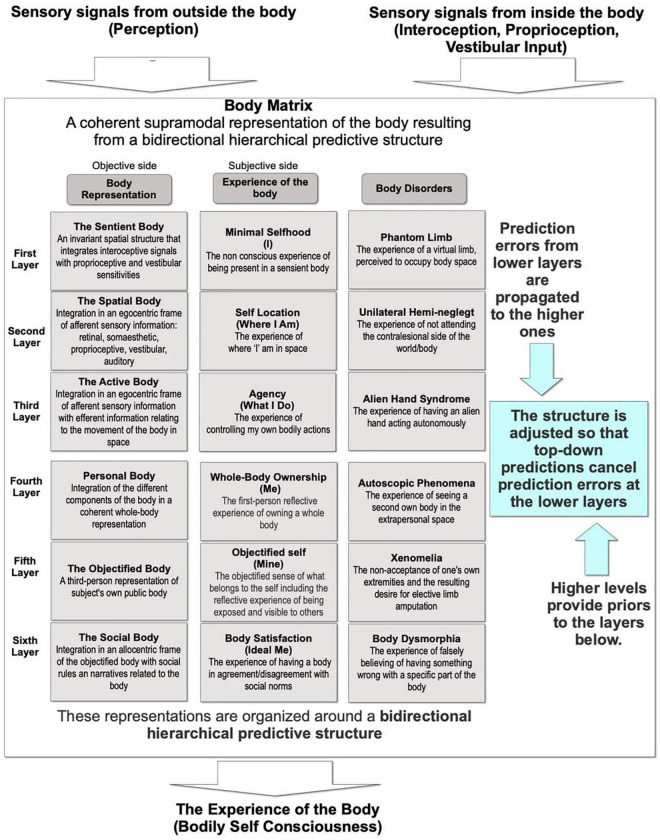
The body matrix.

Even if BSC is experienced by the individual as a unitary perception, neuroimaging and neurological data suggested that BSC includes different layers (Figure 1) that integrate both sensory and cognitive bodily data in a coherent experience (Moseley et al., 2012; Apps and Tsakiris, 2014; Riva, 2018).

Specifically, the layers are organized around a bidirectional hierarchical structure following the active inference and predictive coding paradigms introduced by the Bayesian brain theory (Apps and Tsakiris, 2014; Friston et al., 2014). The result of this process is a coarse supramodal multisensory representation of the body and the space around it (i.e., body matrix, refer to Figure 1), emerging from the flow of information across large-scale networks that link various regions of the brain (Moseley et al., 2012; Gallace and Spence, 2014; Sedda et al., 2016; Riva, 2018).

First, the Bayes’ theorem (Manjaly and Iglesias, 2020) explains how an initial representation/layer (or “prior,” a prediction based on a model of the environment and the body) is integrated with or updated by new observations (i.e., sensory input), resulting in an updated representation (or posterior probability). In this view, the possibility of updating a prior depends on the so-called prediction error, or “surprise” (Stephan et al., 2016): the discrepancy between new data and prior belief (i.e., predicted sensory information), which is weighted by the ratio between data precision (the confidence one assigns to the data) and prior representation precision (the confidence one assigns to a prior belief). In this view, when the precision of the data (i.e., likelihood) is higher than the precision of the prior representation, a large update results i.e., (the posterior moves more strongly toward the data Manjaly and Iglesias, 2020). Simply speaking, precise priors reduce, and precise sensory data increase the probability of a representation update.

Moreover, in a bidirectional hierarchical structure similar to the one used by BSC, higher levels provide priors to the level below, and these constraints are progressively tuned by the sensory input coming from the lower levels (Apps and Tsakiris, 2014; Clark, 2016a). Specifically, prediction errors are propagated to the higher level to adjust the structure of the model so that the top-down predictions cancel prediction errors at the lower level (Clark, 2016a). In this view, the greater is the prediction error at the bottom of the hierarchy (i.e., ascending prediction error), the further up the hierarchy its effects will percolate and lead to the adjustments of the model (Manjaly and Iglesias, 2020). In brief, the minimization of prediction errors involves a reciprocal exchange of signals between hierarchical levels: prediction errors ascend the hierarchy to revise expectations, which generate descending predictions that resolve or suppress prediction errors at the level below.

These principles and different recent studies suggest that it is possible to update the contents of our experience of the body both at a low level, i.e., proprioception and interoception (Henriques and Cressman, 2012; Nourouzpour et al., 2015; Di Lernia et al., 2018), and a high level, i.e., social cognition and self-identification (Tajadura-Jiménez and Tsakiris, 2014; Maister et al., 2015), using advanced technological tools. In the below sections, we further detailed the contents of our proposal: the use of technology-based somatic modification techniques to facilitate a potential revision of maladaptive predictions (priors). Specifically, we planned to use mindful attention, cognitive reappraisal, and brain stimulation techniques merged with high-rewarding and novel synthetic multisensory bodily experiences such as virtual reality bodily illusions.

## Regenerative Virtual Therapy Technology

Since the discovery of the rubber hand illusion (Botvinick and Cohen, 1998) and the emergence of non-invasive brain stimulation methodologies (Tatti et al., 2016), different researchers have used advanced technologies to alter body perceptions in clinical and non-clinical populations. In particular, three different approaches have been developed as follows:

1. **Virtual bodily illusions** (Matamala-Gomez et al., 2021), also known as full-body ownership illusions, use virtual reality technologies to trick the predictive coding mechanisms of the brain, thereby inducing users a sense of ownership over a virtual body.
2. **Interoceptive technologies** (Schoeller et al., 2019), modulate interoceptive signals. They include technologies for producing a direct modulation of interoceptive signals [i.e., c-fibers stimulation, Björnsdotter et al. (2010); Di Lernia et al. (2020); or sonoception, Wiederhold and Riva (2019)] and technologies generating illusions by providing false feedback of the physiological states of individuals (Iodice et al., 2019).
3. **Brain stimulation techniques**, for example, transcranial direct current stimulation (tDCS) and transcranial magnetic stimulation (TMS) (Avenanti et al., 2018; Mancuso et al., 2020; Stramba-Badiale et al., 2020), and also vagus nerve stimulation (Neuser et al., 2020) and galvanic vestibular stimulation (Ponzo et al., 2018, 2019) modify both bottom-up (Pollatos et al., 2016) and top-down (Marotta et al., 2021) bodily signals.

Existing studies, however, suggest that the effects of the abovementioned approaches on higher cognitive processes are temporary, even with non-pathological individuals. For example, as reported by Freeman et al. (2017), the longest follow-up in studies with virtual bodily illusions for correcting the perception of the body in participants with eating disorders is just 2 h (Keizer et al., 2016). In our opinion, this can be explained by the bidirectional hierarchical predictive structure used by BSC. In this structure, the minimization of prediction errors involves a reciprocal exchange of signals between hierarchical layers: prediction errors ascend the hierarchy to revise expectations, which generate descending predictions that resolve or suppress prediction errors at the level below. In this view, generating prediction errors in one layer is not enough to guarantee a revision on higher levels, producing the long-term modification of the BSC.

Following a prediction error, the contents of the body matrix are adjusted in evaluating the (dis)agreement between the perceived sensory activity, and the body experience predicted through the integration of contents from different bodily and cognitive representations (Talsma, 2015). Among others, three possible effects can be activated (Pezzulo et al., 2015; Owens et al., 2018; Mirza et al., 2019), namely, (1) prediction errors ascend the cortical hierarchy to change predictions (model updating), (2) predictions selectively sample sensory input to change the sensations being predicted to agree with their content (active inference) through action and/or attentional shifts, or (3) attention is used to optimize the precision afforded to different parts of the sensorium.

In general, prediction errors in a bidirectional hierarchical predictive structure generate a model updating only when:

– **The extent of the prediction errors is high:** As we have noticed before, the greater is the prediction error, the further up the hierarchy its effects will percolate and lead to model adjustments (Manjaly and Iglesias, 2020). In general, prediction errors arise from the lower layers because they are easier to control and modify. However, errors can be generated in any of the layers of the body matrix (Clark, 2013).
– **The precision of the prediction errors is high:** During multisensory integration, bottom-up bodily signals from different sensory modalities and top-down predictions are weighted according to their contextual reliability and combined to produce a unitary experience of the body. This precision-weighting mechanism is critical for balancing appropriately prediction and sensory stimuli (Barca and Pezzulo, 2020): if it is wrong and it assigns to bottom-up sensory stimuli a low precision, the generated prediction error does not produce an update. Precision operates both within and between modalities, and it is improved both by the level of attention provided to the specific signal (Smout et al., 2019) and by reducing the noise of the sensory signal (Pezzulo et al., 2015).
– **The surprise of the prediction errors is high:** The results of a study by McGuire et al. (2014) suggest that the level of surprise is related to three computationally and neuroanatomically distinct factors.

∘ The first one is the **extent of the prediction error.** The more the outcome is particularly unpredictable or surprising under the current model, the more is the probability of an update.
∘ The second one is **the relative uncertainty of the current model**. The higher is the level of uncertainty of the model, the higher is the probability of an update.
∘ The third one is the level of **reward**. The higher is the potential reward produced by the update, the higher is its probability. Possible rewards are a better image of the self or a positive emotional state.

**FIGURE 2:**
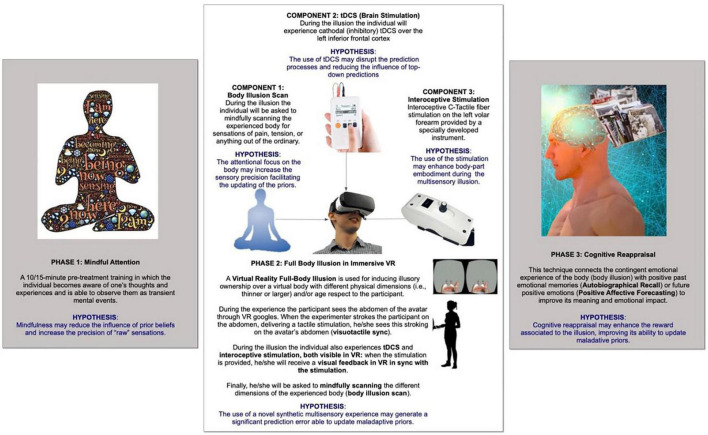
Regenerative virtual therapy.

In this view, the main goal of RVT was to allow a potential revision and de-weighting of maladaptive predictions through the integration of different technology-based somatic modification techniques with mindfulness and cognitive reappraisal. The suggested process is as follows (Figure 2):

1. **The development of a synthetic multisensory experience (visuotactile and interoceptive) able to generate significant prediction errors, for contrasting the dysfunctional internal model**: to reach this goal, we planned to use a virtual reality full-body illusion in a body different from the real one (i.e., an anorectic subject in a normal body) in sync with an interoceptive modulation. During the experience, when the experimenter strokes the participant on the abdomen, delivering a tactile stimulation, he/she observes this stroking on the abdomen of the avatar (visuotactile sync). The same happens during interoceptive stimulation: any stroke on the real hand is synched to the virtual one.
2. **The use of brain stimulation techniques to reduce the influence of top-down predictions**: to manipulate the process of precision-weighting, we suggested the use of tDCS to disrupt the prediction processes and to reduce the influence of top-down predictions. Avenanti et al. used both cathodal (inhibitory) and anodal (excitatory) tDCS over the left inferior frontal cortex, a key area of the action observation network involved in coupling action-perception with execution, during an action prediction task (Avenanti et al., 2018). Their results preliminarily suggest that down- and upregulating excitability using tDCS can hinder and enhance action prediction abilities, respectively.
3. **The use of mindfulness attention to improving the precision of the synthetic multisensory experience:** mindful attention (Papies et al., 2015) is defined as a form of attention that can increase the salience of the moment-by-moment experience and reduce the impact of predefined schemas. When mindfully attentive, people become aware of thoughts and experiences, observing them as transient mental events (Bennett et al., 2021). Mindful attention is a primary component of mindfulness, which can be considered the awareness of being in the present moment without the burden of previous, mindless schemas (Pagnini and Philips, 2015). In Bayesian terms, this suggests that mindfulness may reduce the influence of prior beliefs and increase the precision of “raw” sensations (Manjaly and Iglesias, 2020). Preliminary findings supporting this hypothesis suggest that automatic reactions and behaviors (Papies et al., 2015), such as salivation following food conditioning (Baquedano et al., 2017), can be hindered with mindful attention.
4. **The use of cognitive reappraisal to reconstruct and re-elaborate the emotional content of the multisensory experience to improve its level of reward:** specifically, we planned to increase the level of reward by connecting the body illusion with positive past emotional memories [i.e., autobiographical recall (AR)] or future positive emotions [i.e., positive affective forecasting (PAF)] to improve its meaning and emotional impact. AR connects the contingent emotional experience of the body with past emotional memories of it (Robinson, 1986; Mills and D’Mello, 2014). Instead, PAF connects the body illusion to how the individual will feel in the future.

## Conclusion

This perspective introduced the RVT, a new therapeutical approach that wants to address a critical feature of most, if not all, psychopathologies: schema rigidity (Morris and Mansell, 2018). According to a predictive brain neuroscientific approach, mental disorders can be broadly characterized by a dysfunction in the way the brain computes and integrates the representations of the inner and outer body across time (i.e., BSC). Specifically, inaccurate or inflexible predictions can disturb the coherent integration of bodily and visceral signals and disrupt the optimal interaction of an individual with the external and social world.

In this view, the main goal of RVT was to allow a potential revision and de-weighting of maladaptive predictions through the integration of different technology-based somatic modification techniques with mindfulness and cognitive reappraisal.

The perspective discussed the rationale of this approach and presented a specific strategy based on the following steps:

– The development of a synthetic multisensory experience (i.e., visuotactile and interoceptive) to generate significant prediction errors: a virtual reality full-body illusion in sync with an interoceptive modulation characterized by a low level of predictability.
– The use of brain stimulation techniques to reduce the influence of top-down predictions.
– The use of mindfulness attention to improving the precision of the multisensory experience.
– The use of cognitive reappraisal to reconstruct and re-elaborate the emotional content of the multisensory experience to improve its level of reward.

On the one hand, this framework is based on a clear rationale and allows the identification of different hypotheses (presented in Figure 2) that can be tested experimentally. On the other hand, the clinical testing of the different assumptions is not easy, not only experimentally but also technically and computationally. The biggest challenge is the complexity of the different multisensory bodily experiences to be developed that involve both internal and external signals and both somatic and semantic/metacognitive domains.

Moreover, the closed-loop nature of BSC means that a modification in one domain typically invokes a cascade of changes throughout the different layers, making it difficult to differentiate cause from consequence. This suggests that, on the one hand, it is complex to evaluate the effects of the treatment given the many variables involved. On the other hand, the regulation of bodily variables through homeostasis and allostasis makes particularly challenging to determine whether problems of a patient originate in inference problems, regulation problems, or actual bodily dyshomeostasis as these can all lead to one another (Petzschner et al., 2017).

Finally, the number of somatic perturbation techniques that can be used to generate prediction errors is actually limited to the ones described in the study. As underlined by Petzschner et al. (2017), developing new tools that are non-invasive and provide temporal control is critical for the future of RVT. Moreover, the increasing availability of tools that allow the acquisition and (computational) analysis of neuroimaging and behavioral data may facilitate the validation of the model (Frässle et al., 2021).

In conclusion, RVT offers an empirically testable and potentially clinically useful framework that can improve the existing state-of-the-art in different ground-breaking ways, allowing us as follows:

– to acquire an unprecedented experimental overview of the dynamics of our bodily representations.
– to explain how somatic processes affect mental health and wellbeing.
– to reverse-engineer their functioning and hacking them using interoceptive and multisensory technologies.

## Data Availability Statement

The original contributions presented in the study are included in the article/supplementary material, further inquiries can be directed to the corresponding author/s.

## Author Contributions

GR conceived and developed the initial draft. FP revised the initial draft. SS, DD, and FP worked with GR to enhance the revised draft and develop it into the final draft. All authors have reviewed and approved the final manuscript as submitted.

## Conflict of Interest

The authors declare that the research was conducted in the absence of any commercial or financial relationships that could be construed as a potential conflict of interest.

## Publisher’s Note

All claims expressed in this article are solely those of the authors and do not necessarily represent those of their affiliated organizations, or those of the publisher, the editors and the reviewers. Any product that may be evaluated in this article, or claim that may be made by its manufacturer, is not guaranteed or endorsed by the publisher.

## Abbreviations

AR: autobiographical recall
BSC: bodily self-consciousness
GVS: galvanic vestibular stimulation
PAF: positive affective forecasting
RCT: randomized controlled trial
RVT: regenerative virtual therapy
tDCS: transcranial direct current stimulation
TMS: transcranial magnetic stimulation
VNS: vagus nerve stimulation.
